# Foot posture influences the electromyographic activity of selected lower limb muscles during gait

**DOI:** 10.1186/1757-1146-2-35

**Published:** 2009-11-26

**Authors:** George S Murley, Hylton B Menz, Karl B Landorf

**Affiliations:** 1 Department of Podiatry, Faculty of Health Sciences, La Trobe University, Bundoora, Australia; 2 Musculoskeletal Research Centre, Faculty of Health Sciences, La Trobe University, Bundoora, Australia

## Abstract

**Background:**

Some studies have found that flat-arched foot posture is related to altered lower limb muscle function compared to normal- or high-arched feet. However, the results from these studies were based on highly selected populations such as those with rheumatoid arthritis. Therefore, the objective of this study was to compare lower limb muscle function of normal and flat-arched feet in people without pain or disease.

**Methods:**

Sixty adults aged 18 to 47 years were recruited to this study. Of these, 30 had normal-arched feet (15 male and 15 female) and 30 had flat-arched feet (15 male and 15 female). Foot posture was classified using two clinical measurements (the arch index and navicular height) and four skeletal alignment measurements from weightbearing foot x-rays. Intramuscular fine-wire electrodes were inserted into tibialis posterior and peroneus longus under ultrasound guidance, and surface EMG activity was recorded from tibialis anterior and medial gastrocnemius while participants walked barefoot at their self-selected comfortable walking speed. Time of peak amplitude, peak and root mean square (RMS) amplitude were assessed from stance phase EMG data. Independent samples *t*-tests were performed to assess for significant differences between the normal- and flat-arched foot posture groups.

**Results:**

During contact phase, the flat-arched group exhibited increased activity of tibialis anterior (peak amplitude; 65 versus 46% of maximum voluntary isometric contraction) and decreased activity of peroneus longus (peak amplitude; 24 versus 37% of maximum voluntary isometric contraction). During midstance/propulsion, the flat-arched group exhibited increased activity of tibialis posterior (peak amplitude; 86 versus 60% of maximum voluntary isometric contraction) and decreased activity of peroneus longus (RMS amplitude; 25 versus 39% of maximum voluntary isometric contraction). Effect sizes for these significant findings ranged from 0.48 to 1.3, representing moderate to large differences in muscle activity between normal-arched and flat-arched feet.

**Conclusion:**

Differences in muscle activity in people with flat-arched feet may reflect neuromuscular compensation to reduce overload of the medial longitudinal arch. Further research is required to determine whether these differences in muscle function are associated with injury.

## Background

Human foot posture is highly variable among healthy individuals and ranges from flat- to high-arched [1]. While foot posture is strongly influenced by some systemic conditions, such as neurological and rheumatological diseases, there is emerging evidence that variations in foot posture among healthy individuals are associated with changes in lower limb motion [2,3], and in some cases, increased risk of lower limb injury [4,5]. The link between variations in foot posture and increased risk of lower limb injury may arise from abnormal muscle activity. For example, it has been suggested that the flat-arched foot relies on additional muscular support during gait [2], and that fatigue of these controlling muscles with exercise can result in the development of various injuries such as tibial stress fractures [6].

With this mind, we recently conducted a systematic review of studies that investigated the effect of foot posture on lower limb muscle activity during walking or running [7]. The review concluded that there is some evidence to indicate that pronated foot posture is associated with greater electromyography (EMG) amplitude for invertor muscles, such as tibialis posterior, and lower EMG amplitude for evertor muscles, such as peroneus longus, when compared to normal or supinated foot posture. However, these findings may not be generaliseable to the wider population because of highly selected samples. For instance, the best evidence to date that indicates tibialis posterior muscle activation is greater in flat-arched foot posture was reported by a study comprising older adults with long-standing rheumatoid arthritis [8]. Therefore, other than the early descriptive work of Gray and Basmajian in 1968 [9], it is unknown whether foot posture influences tibialis posterior muscle activation in adults without pain or dysfunction.

Another issue with previous studies is that strategies for classifying foot posture have infrequently included valid and reliable measurements. Several methods of classifying foot posture have been employed, including: the arch index [10], the arch ratio [11], radiographic alignment [8], two-dimensional video analysis [12] and subjective clinical observation [2,9]. Furthermore, only in the last decade has normative foot posture data for various clinical and radiological measurements been published [3,13-16]. Utilising these data, we recently developed a protocol for classifying foot posture based on both clinical and radiographic measurements [16]. We hypothesised that adopting a more systematic approach to classifying foot posture would assist in the identification of functional differences in EMG activity between foot types.

With these issues in mind, the objective of this study was to investigate EMG activity of tibialis posterior, peroneus longus, tibialis anterior and medial gastrocnemius in healthy adults with normal- and flat-arched foot posture.

## Methods

### Participants

**Figure 1:**
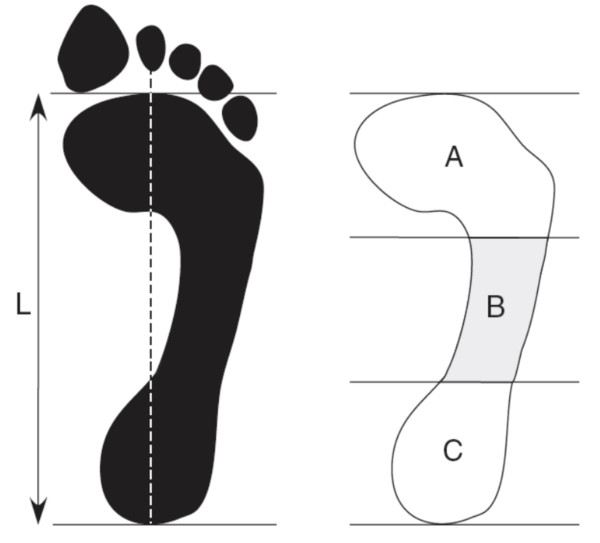
**Footprint with reference lines for calculating the arch index**. The length of the foot (excluding the toes) is divided into equal thirds to give three regions: A -- forefoot; B -- midfoot; and C -- heel. The arch index is then calculated by dividing the midfoot region (B) by the entire footprint area (i.e. Arch index = B/[A+B+C]).

**Figure 2:**
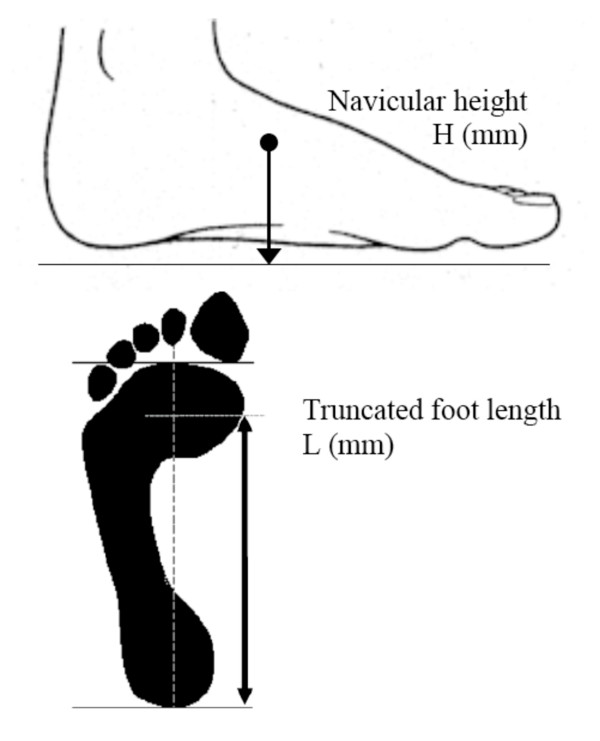
**Calculating normalised navicular height truncated**. The distance between the supporting surface and the navicular tuberosity is measured. Foot length is truncated by measuring the perpendicular distance from the 1^st ^metatarsophalangeal joint to the most posterior aspect of the heel. Normalised navicular height truncated is calculated by dividing the height of the navicular tuberosity from the ground (H) by the truncated foot length (L) (i.e. Normalised navicular height truncated = H/L).

**Figure 3:**
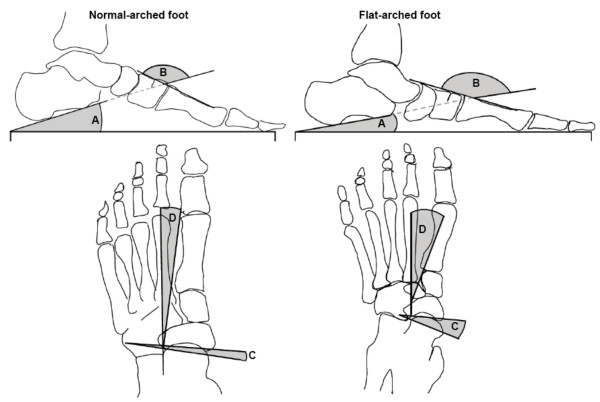
**Traces from two representative participants illustrate x-ray angular measurements from normal (left) and flat-arched (right) foot posture**. Lateral views (top) show: calcaneal inclination angle; calcaneal-first metatarsal angle; anterior posterior views (bottom) show: talonavicular coverage angle; talus second metatarsal angle. A - calcaneal inclination angle, B - calcaneal-first metatarsal angle, C - talo-navicular coverage angle, D - talus-second metatarsal angle. Angle A *decreases *with flat-arched foot posture; angle B, C and D *increase *with flat-arched foot posture, compared to the normal-arched foot posture.

Sixty adults aged 18 to 47 years were recruited to this study. Of these, 30 had normal-arched feet (15 male and 15 female) and 30 had flat-arched feet (15 male and 15 female). Participant characteristics are presented in Table 1. A foot screening protocol that included both clinical and radiographic measures to classify foot posture was used to recruit participants with normal- and flat-arched feet [16]. This protocol was derived from normative foot posture values for two clinical measurements (the arch index and navicular height) and four angular measurements obtained from antero-posterior and lateral x-rays (talus-second metatarsal angle, talonavicular coverage angle, calcaneal inclination angle and calcaneal-first metatarsal angle) [16]. To qualify for the normal-arched foot group, participants had either a normal arch index or navicular height measurement, and their four radiographic measurements were within a normal range. To qualify for the flat-arched group, participants had an arch index or navicular height measurement greater than two standard deviations from mean values obtained for the normal-arched group. Furthermore, their radiographic measurements were greater than 1 standard deviation from the mean values obtained for the normal-arched group for either the sagittal and or transverse plane measurements. Figures 1, 2 and 3 illustrate the clinical and radiographic measurements.

**Table 1:** Participant characteristics

	Foot posture groups	
	
	Flat-arch n = 30	Normal-arch n = 30
**General anthropometric**		
Gender ratio (female/male)	15/15	15/15
Age mean ± SD (years)	21.8 ± 4.3	23.6 ± 5.9
Height mean ± SD (cm)	171.0 ± 10.0	169.7 ± 9.7
Weight mean ± SD (Kg)	73.3 ± 15.50	69.9 ± 13.6
Left or right foot count^FC^	13 right/17 left	13 right/17 left
**Clinical measurements**		
AI mean ± SD	0.30 ± 0.07*	0.24 ± 0.04*
NNHt mean ± SD	0.18 ± 0.04^†^	0.27 ± 0.03^†^
**Radiographic measurements**		
CIA mean ± SD (degrees)	15.7 ± 4.5^#^	20.8 ± 3.5^#^
C1MA mean ± SD (degrees)	142.3 ± 6.0^‡^	132.8 ± 4.1^‡^
TNCA mean ± SD (degrees)	27.6 ± 9.0^	11.9 ± 8.1^
T2MA mean ± SD (degrees)	27.1 ± 10.1^¥^	13.0 ± 6.4^¥^
**Walking velocity**	1.21 ± 0.13**	1.10 ± 0.11**

AI -- arch index, NNHt -- normalised navicular height truncated, CIA -- calcaneal inclination angle, C1MA -- calcaneal first metatarsal angle, TNCA -- talo-navicular coverage angle, T2MA -- talus-second metatarsal angle.^FC^denotes the number of participants whose left or right foot was suitable for inclusion in their respective group (i.e. normal-arch or flat-arch).

Mean differences and 95% confidence interval (CI) expressed relative to normal-arch.

Statistically significant findings for comparisons listed below (*p *< 0.001):

* AI: mean difference 0.06, 95% CI 0.03 to 0.09

^† ^NNHt: mean difference -0.09, 95% CI -0.11 to -0.08

^#^CIA: mean difference -5.13°, 95% CI -7.21 to -3.05°

^‡^C1MA: mean difference 9.47°, 95% CI 6.8 to 12.14°

^TNCA: mean difference 15.70°, 95% CI 11.28 to 20.12°

^¥ ^T2MA: mean difference 14.08°, 95% CI 9.73 to 18.44°

** Walking speed: mean difference 0.11 ms, 95% CI 0.05 to 0.17 ms

The participants were without symptoms of macrovascular disease (e.g. angina, stroke, peripheral vascular disease), neuromuscular disease, or any biomechanical abnormalities that affected their ability to walk. Ethical approval was obtained for the study from the La Trobe University Human Ethics Committee (Ethics ID: FHEC06/205) and the study was registered with the Radiation Safety Committee of the Victorian Department of Human Services. The x-rays were performed in accordance with the Australian Radiation Protection and Nuclear Safety Agency Code of Practice for the Exposure of Humans to Ionizing Radiation for Research Purposes (2005) [17].

### Experimental protocol

Bipolar fine-wire intramuscular electrodes were used to record the EMG signal from tibialis posterior and peroneus longus. The electrodes were fabricated from 75 μm Teflon^® ^coated stainless steel wire (A-M Systems, Washington, USA) with 1 mm of insulation stripped to form the recording surface of the two wires. The electrode wires were inserted into a 23 gauge sterilized single use hypodermic needle with the exposed electrode tips bent 3 mm and 5 mm to prevent the contact areas from touching during recording. The process of fine-wire electrode construction and positioning of wires in vivo was undertaken in accordance with previous work [14] (Additional file 1).

Tibialis anterior and medial gastrocnemius EMG was recorded with the use of DE-3.1 surface electrodes (Delsys Inc., Boston, USA). The electrodes featured a double differential 3-bar type configuration with a 99.9% silver contact material and an inter-electrode distance of 10 mm. The application of surface electrodes followed the recommendations of SENIAM [18].

The temporal characteristics of the walking cycle were measured using circular force sensitive resistors (footswitches) with a diameter of 13 mm (Model: 402, Interlink Electronics, California, USA). These were placed on the plantar surface of the interphalangeal joint of the hallux and the most posterior plantar aspect of the calcaneus to record the timing of heel contact, toe contact, heel off and toe off.

During testing, participants were instructed to walk at their self-selected walking speed, which was established following a warm-up period from two trials along a 9 m walkway. Six trials were recorded during testing, with any trial exceeding ± 5% of the average warm-up speed excluded and subsequently repeated.

Maximum voluntary isometric contractions (MVIC) were used for normalising EMG amplitude parameters. At the completion of each testing session, three MVICs for each muscle were undertaken comprised of a gradual and continuous 2 s build-up followed by a maximum 2 s effort. Each participant was instructed to perform a maximum contraction against the resistance of the tester and was given verbal encouragement while doing so. The resisted movements included; supination - tibialis posterior, pronation - peroneus longus, dorsiflexion - tibialis anterior, plantarflexion (knee extended) - medial gastrocnemius. The participant sat on a bench while performing the MVICs for tibialis posterior, tibialis anterior and the peroneal muscles. For the medial gastrocnemius MVICs, the participant sat on the floor with their back against a wall, to ensure the participant did not slide backward during the contraction.

Three consecutive maximum efforts were separated by a 1 min recovery period. A 600 ms window in the middle of the 2 s recording period was used to calculate average root mean square (RMS) from three trials.

### Data processing

During the gait trials, the raw EMG signal was passed through a differential amplifier at a gain of 1000 with a sampling frequency of 2 kHz. A band pass filter (built into the amplifier; Delsys Inc., Boston, USA) of 20-2000 Hz was applied to the intramuscular electrodes and 20-450 Hz for the surface electrodes.

**Figure 4:**
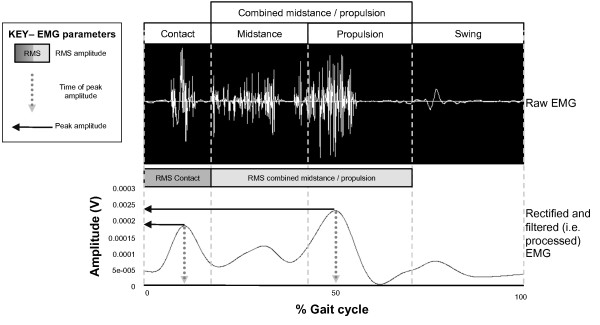
**A single gait cycle showing raw and processed EMG for tibialis posterior from a single participant**. Time of peak amplitude, peak amplitude and RMS amplitude (root mean square) were derived from the linear envelope (processed curve).

EMG data from the MVICs and walking trials were full wave rectified and low pass filtered at a cut off frequency of 6 Hz through a 4^th ^order Butterworth filter with phase lag. Data were analysed from the third or fourth stride depending on the quality of the footswitch signal. Two consecutive strides were analysed for each trial and averaged from the last four of six trials for each speed (i.e. four average gait cycles derived from eight ipsilateral steps). Three EMG parameters were analysed for each muscle, including: (i) time of peak amplitude; (ii) root mean square (RMS); and (iii) peak amplitude (Figure 4). These parameters have been utilised in previous single-session investigations [14,19,20]. The following phases of the gait cycle were assessed based on when these muscles are most active in normal-arched feet [14]: tibialis posterior and peroneus longus - contact and combined midstance/propulsion phase; tibialis anterior - contact phase; and medial gastrocnemius - combined midstance/propulsion phase.

### Statistical analysis

The distribution of data was evaluated from skewness and kurtosis values and Levene's test for equality of variances. Independent samples *t*-tests were performed to assess for significant differences between the normal- and flat-arched groups for anthropometric characteristics, walking speed and EMG parameters with *p *values less than 0.05 considered significant.

## Results

### Participant characteristics

The normal- and flat-arched foot posture groups were matched for age, gender, height and weight, with no significant differences for any of these characteristics except for the clinical and radiographic measures of foot posture (Table 1). However, the self-selected comfortable walking speed of the flat-arched group was slightly greater than the normal-arched group (mean difference: 0.11 ms, 95% CI: 0.05 to 0.17, *p *< 0.001).

### Effect of foot posture on muscle EMG activation

Comparisons of EMG variables between the normal- and flat-arched foot groups are presented in Table 2. Statistically significant differences in peak and RMS EMG amplitude were detected for tibialis posterior, peroneus longus and tibialis anterior. There were no significant differences in EMG time of peak amplitude.

**Table 2:** Effect of foot posture on all EMG variables

Muscle	Phase of gait cycle	EMG parameter	% mean difference ^	95% CI	Effect size ^#^	*p *value (2-tailed)
Tibialis posterior	Contact	TimePeak	0.1	0.0 to 1.7	0.52	0.051
		PeakAmp	-14.3	-29.1 to 0.4	0.51	0.058
		RMS	-7.8	-18.4 to 2.7	0.39	0.144
	Mid/Prop	TimePeak	0.0	-3.8 to 3.7	0.01	0.980
		PeakAmp	26.5*	4.2 to 48.7	0.69	0.021*
		RMS	16.4*	3.6 to 29.1	0.68	0.013*

Peroneus longus	Contact	TimePeak	1.6	0.0 to 3.2	0.51	0.057
		PeakAmp	-12.8*	-25.1 to -0.5	0.48	0.041*
		RMS	-6.6	-13.9 to 0.6	0.48	0.075
	Mid/Prop	TimePeak	3.3	-0.3 to 6.9	0.47	0.079
		PeakAmp	-20.0	-42.9 to 2.9	0.46	0.086
		RMS	-13.7*	-26.1 to -1.4	0.58	0.030*

Tibialis anterior	Contact	TimePeak	0.1	-0.7 to 0.9	0.09	0.737
		PeakAmp	19.0*	11.2 to 26.9	1.3	<0.001*
		RMS	10.4*	4.0 to 16.8	0.87	0.002*

Medial oastrocnemius	Mid/Prop	TimePeak	0.4	-1.8 to 2.7	0.10	0.715
		PeakAmp	2.7	-15.4 to 20.7	0.12	0.766
		RMS	7.2	-12.3 to 16.9	0.22	0.753

Contact -- contact period of gait cycle; Mid/Prop -- combined midstance and propulsion period of gait cycle; TimePeak -- time of peak amplitude; PeakAmp -- peak EMG amplitude; RMS -- root mean square amplitude; ^ relative to normal-arch foot group; CI -- confidence interval;^# ^Cohen's *d *calculation;

* statistically significant independent sample *t*-test (*p *< 0.05)

#### Contact phase - heel contact to toe contact

**Figure 5:**
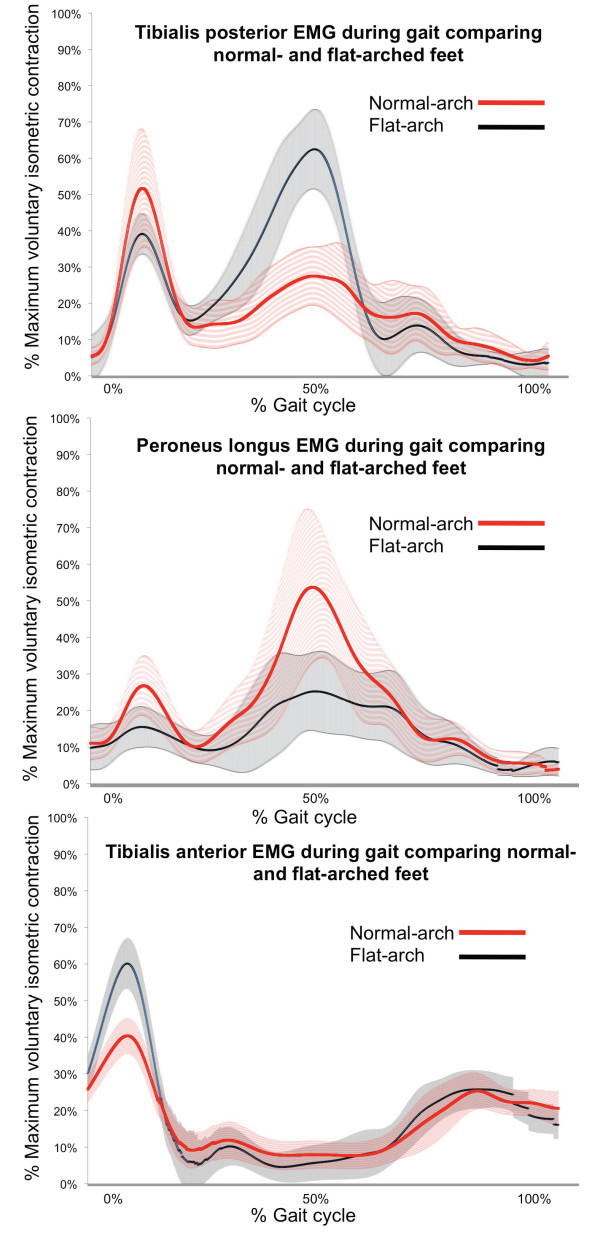
**Ensemble averaged EMG curves for tibialis posterior, peroneus longus and tibialis anterior for 30 participants with normal-arch and 30 participants with flat-arch feet**. The curves differ slightly to the actual results (Table 2), as these curves are derived from a single gait cycle for each participant to illustrate the main findings. Solid lines -- mean amplitude; shaded area surrounding solid line -- 95% confidence interval. Significant differences are generally indicated where 95% confidence intervals separate between groups. HC - heel contact.

For tibialis anterior, the flat-arched group exhibited increased peak EMG amplitude (mean difference: 19.0%; 95% CI: 11.2 to 26.9; *d = 1.3; p *< 0.001) and RMS amplitude (mean difference: 10.4%; 95% CI: 4.0 to 16.8; *d = 0.87; p *= 0.002), compared to the normal-arched group. For peroneus longus, the flat-arched foot group exhibited decreased peak EMG amplitude (mean difference: -12.8%; 95% CI: -25.1 to -0.5; *d = 0.48; p *= 0.041), compared to the normal-arched group (Figure 5). For tibialis posterior, the flat-arched foot group exhibited decreased peak EMG amplitude (mean difference: -14.3%; 95% CI: -29.1 to 0.4; *d = 0.51; p *= 0.058) compared to the normal arched group, although this finding did not reach statistical significance (Figure 5).

#### Midstance/propulsion phase - toe contact to toe-off

For peroneus longus, the flat-arched foot group exhibited decreased peak EMG (mean difference: -13.7%; 95% CI: -26.1 to -1.4; *d = 0.58; p *= 0.030), compared to the normal-arched group (Figure 5). For tibialis posterior, the flat-arched group exhibited increased peak EMG amplitude (mean difference: 26.5%; 95% CI: 4.2 to 48.7; *d = 0.69; p *= 0.021) and RMS amplitude (mean difference: 16.4%; 95% CI: 3.6 to 29.1; *d = 0.68; p *= 0.013), compared to the normal-arched group (Figure 5). No significant differences between groups were detected for medial gastrocnemius.

## Discussion

The objective of this study was to investigate the effect of flat-arched foot posture on the EMG activity of selected leg muscles. During comfortable walking, participants in the flat-arched foot group functioned at a significantly greater percentage of their maximum amplitude for tibialis posterior during midstance/propulsion phase, compared to participants in the normal-arched group (peak amplitude, 86 versus 60% of MVIC; RMS amplitude, 48 versus 31% of MVIC). Similar trends have been reported by earlier studies comparing these foot types [8,9], however these studies did not report 95% confidence intervals for the percentage difference or effect size calculations, making it difficult to assess the precision and the magnitude of the differences observed [7]. Effect sizes for the differences observed in peak and RMS for tibialis posterior amplitude were 0.68 and 0.69 respectively, representing moderate differences in muscle activity. Despite the issue of random variability for tibialis posterior EMG amplitude during gait [14,20], our results provide strong evidence to indicate that tibialis posterior is working harder (i.e. as a percentage of a maximum contraction) during midstance/propulsion in participants with flat-arched feet, compared to those with normal-arched feet.

One explanation for our findings is that the medial longitudinal arch and supportive structures (e.g. ligaments) of a flat-arched foot may undergo greater loading during walking, compared to the normal-arched foot. Greater loading of the medial arch would require greater work from tibialis posterior to protect the arch structures from excessive tissue stress and injury. While cadaveric research has shown an increased loading of the foot's medial structures with simulated tibialis posterior tendon dysfunction [21], it is also possible that these events can occur in reverse, that is, the flat-arched foot may place a greater demand on tibialis posterior. This mechanism is further supported by our findings for peroneus longus.

In contrast to tibialis posterior, participants in the flat-arched group functioned at a significantly lower percentage of their maximum amplitude for peroneus longus during contact phase and midstance/propulsion phase, compared to participants in the normal-arched group (peak amplitude - contact phase, 24 versus 37% MVIC; RMS amplitude - midstance/propulsion, 25 versus 39% MVIC). These findings indicate that peroneus longus is working less during the contact and midstance/propulsion phases in participants with flat-arched feet, compared to those with normal-arched feet. Effect sizes for these differences were 0.48 and 0.58 for peak amplitude (contact phase) and RMS (midstance/propulsion phase) amplitude respectively, representing moderate differences in muscle activity. These functional differences between foot types may reflect a compensatory lack of activity in peroneus longus to avoid further overloading the medial arch. Alternatively, this finding may occur as a result of flat-arched feet being less laterally unstable, therefore requiring less peroneus longus activity.

A further significant finding was that participants in the flat-arched group functioned at a significantly greater percentage of their maximum amplitude for tibialis anterior during contact phase, compared to participants in the normal-arched group (peak amplitude, 65 versus 46% MVIC; RMS amplitude, 43 versus 32% MVIC). Effect sizes for these differences were 1.3 and 0.87 for peak and RMS amplitude respectively, representing large differences in muscle activity. During contact phase of the gait cycle, tibialis anterior is thought to decelerate ankle joint plantarflexion and resist foot pronation [22]. Interestingly, the role of tibialis anterior in resisting pronation of the foot during the contact phase was not assisted via strong co-activation of tibialis posterior. In fact, tibialis posterior functioned at a lower percentage amplitude during contact phase compared to the normal arched group, although this finding did not reach statistical significance (*p *= 0.058).

There were no differences in medial gastrocnemius timing or amplitude EMG parameters comparing normal- and flat-ached feet. This finding adds to the growing body of evidence that medial gastrocnemius muscle activation is not affected by differences in foot posture [7]. Furthermore, this indicates that medial gastrocnemius is unlikely to have a significant function as an inverter of the hindfoot, since deviations in hindfoot alignment have not been shown to cause changes in the activity of this muscle [7].

The finding that participants in the flat-arched foot group walked slightly faster than those in the normal-arched group (mean difference, 0.11 ms) was unexpected and may have influenced some results in this study. It should be noted that both foot posture groups were instructed to walk at their normal comfortable walking speed and data collection was carried out under identical conditions. This difference in walking speed required some consideration, as numerous studies investigating the influence of walking speed on EMG amplitude have indicated that EMG amplitude increases linearly with walking speed [23-25]. There may be a biological or compensatory reason why participants with flat-arched feet walked faster than those with normal-arched feet, such as a means of increasing stability of the foot and lower limb during walking. In this case, the independent variable (flat-arch foot posture) may have influenced the covariate (walking speed), and this poses a conceptual issue preventing us from adopting an analysis of co-variance approach to adjust for walking speed [26]. However, we believe that the differences in muscle activity observed between the groups are unlikely to have been caused by differences in walking speed. Participants in the flat-arch group functioned at a significantly *lower *percentage of their maximum amplitude for peroneus longus during contact phase and midstance/propulsion phase, despite walking faster. Furthermore, den Otter and colleagues [23] have shown that negative amplitude gains (i.e. increased amplitude with reduced walking speed) of peroneus longus only occur at very slow speeds. Therefore, it is unlikely that the normal-arched group displayed a relative 'negative gain' compared to the flat-arched group.

The results presented here may have implications for the management of lower extremity overuse conditions. Although it is still unknown whether these functional differences in muscle activation are beneficial or detrimental in relation to injury, preliminary evidence indicates that these differences may be reversible with intervention [27]. In a recent study, Franettovich and colleagues [27] investigated the effect of an anti-pronation taping technique on lower limb EMG muscle activation in four adults with pronated foot posture. They reported that the anti-pronation tape significantly reduced the EMG amplitude of the tibialis posterior and tibialis anterior muscles during walking. While this indicates that anti-pronation tape may bring muscle function in a flat-arched foot closer to that observed in a normal-arched foot, further research is required to ascertain whether these changes are associated with clinical outcomes.

This study has several strengths, including the use of a rigorous protocol to classify foot posture, the use of in-dwelling needle electrodes to assess tibialis posterior and peronus longus, and a relatively large sample size (n = 60 compared to 17 to 43 in previous studies [2,7-10,12]). However, the results of this study also need to be interpreted in light of two limitations. Firstly, we did not simultaneously record other kinematic and kinetic variables, thus we can only speculate as to the mechanical effects of the EMG differences. Secondly, the participants in this study were relatively homogenous as they were mostly young, healthy and without musculoskeletal injury. Therefore, caution should be taken in generalising these results to symptomatic or clinical populations. A further limitation was that we used MVICs to normalise the EMG amplitude parameters. It is difficult to control and monitor the participants' effort or output with MVICs which may be a factor that leads to greater between-participant variability compared to other normalisation protocols [20].

## Conclusion

Lower limb muscle function is affected by foot posture. The flat-arched group functioned at a greater percentage of their maximum EMG amplitude during contact phase for tibialis anterior and during midstance/propulsion for tibialis posterior, compared to normal-arched feet. The flat-arched foot group also functioned at a lower percentage of their maximum EMG amplitude throughout stance phase for peroneus longus, compared to normal-arched feet. These differences in muscle activity may reflect neuromuscular compensation to reduce overload of the medial longitudinal arch in people with flat-arched feet. Further research is required to determine whether these differences in muscle function are associated with injury.

## Competing interests

HBM and KBL are Editor-in-Chief and Deputy Editor-in-Chief, respectively, of *Journal of Foot and Ankle Research*. It is journal policy that editors are removed from the peer review and editorial decision-making processes for papers they have co-authored.

## Authors' contributions

GSM, HBM and KBL conceived the idea and obtained funding for the study. GSM, HBM and KBL designed the study protocol. GSM recruited participants, conducted the laboratory testing and processed data. GSM, HBM and KBL drafted the manuscript. All authors have read and approved the final manuscript.

## Supplementary Material

Additional file 1 A video demonstration of the insertion of an intramuscular electrode into tibialis posterior via the posterior approach Click here for file
